# Editorial: Dialysis and the age-friendly health system initiative

**DOI:** 10.3389/fneph.2023.1170566

**Published:** 2023-03-14

**Authors:** Dipal M. Patel, C Barrett Bowling, Laura C. Plantinga, Bernard G. Jaar

**Affiliations:** ^1^ Division of Nephrology, Department of Medicine, Johns Hopkins University, Baltimore, MD, United States; ^2^ Durham Veterans Affairs Geriatric Research Education and Clinical Center, Durham Veterans Affairs Health Care System (VAHCS), Durham, NC, United States; ^3^ Department of Medicine, Emory University School of Medicine, Atlanta, GA, United States; ^4^ Department of Epidemiology, Johns Hopkins Bloomberg School of Public Health, Baltimore, MD, United States; ^5^ Welch Center for Prevention, Epidemiology, and Clinical Research, Johns Hopkins Bloomberg School of Public Health, Baltimore, MD, United States

**Keywords:** end-stage kidney disease, dialysis, patient-centered care, older adults, age-friendly health system

Older patients requiring dialysis are at high risk of morbidity and mortality, with an average of less than three expected remaining years of life in prevalent patients with kidney failure aged 85 years or older (1). In fact, in patients over 75 years old with comorbidities, initiation of dialysis may not confer a large survival advantage compared to conservative management (2). Still, patients over 75 years old comprise the largest age group of prevalent patients on dialysis (1). To engage in well-informed shared decision-making surrounding treatment options, clinicians should possess adequate knowledge surrounding the symptom burden and life impact of kidney disease in older patients. Acknowledgement, assessment, and management of geriatric syndromes is highly relevant to care of this vulnerable group of patients.

The John A. Hartford Foundation and Institute for Healthcare Improvement have advocated for construction of the Age-Friendly Health System, which reinforces inclusion of the “4Ms” as a means to providing high-quality patient-centered care to older adults (3). The 4Ms include: 1) mentation (prevention, identification, and management of cognitive impairment, mental health conditions, and delirium); 2) mobility (maintenance of function and safe movement); 3) what matters (aligning with patient-determined goals and preferences); and 4) medication (using medications only when needed, and avoiding medications which interfere with other elements of the 4Ms). Here, we highlight five recent articles pertinent to the 4Ms as part of the special Research Topic, “*Dialysis and the age-friendly health system initiative”*.

First, what are the current practices in caring for older patients with chronic kidney disease (CKD)? By utilizing data from two patient cohorts [the French Renal Epidemiology and Information Network (REIN) cohort, which has been extended to include patients with CKD stages 4 and 5 since 2019, and the Parcours de Soins des Personnes Agées (PSPA) cohort of patients aged > 75 years with advanced CKD], Moranne et al. identify several key issues in the care of this patient subgroup. In their analysis, 77% of patients in the PSPA cohort were prescribed medications that were deemed to be “renally inappropriate,” and fewer than half of patients aged ≥75 years with CKD stage 5 had received education about kidney failure treatment options. Quality of life scores were lower for patients on dialysis, compared to those who had received a kidney transplant or remained off of dialysis. Fitting with the Age-Friendly Health System Initiative, the authors call for patient-centered discussions of the risk and benefit of each medication and for shared decision-making while planning for potential renal replacement therapy.

Next, when longitudinally caring for patients who experience a decline in kidney function and/or receipt of renal replacement therapy, are clinicians equipped with enough support to monitor anticipated life changes and symptom burden? Though some patient-reported outcomes measures such as the Kidney Disease Quality of Life (KDQOL) (4) are used in care of patients on dialysis, patients with kidney disease may place higher value in specific symptoms or outcomes which have not traditionally been quantified. Mobility, for instance, was rated as a key outcome of importance by patients on hemodialysis (5) but may often go unmonitored. To measure physical activity, which draws upon patient-centered outcomes of mobility and resilience, Lucas et al. demonstrate feasibility of using accelerometers in older patients receiving hemodialysis. Of 37 participants receiving outpatient hemodialysis, 29 [mean age 70.6 (S.D. 4.8) years] were able to submit sufficient accelerometer data, which demonstrated that step count variability correlated with measures of physical function. Distribution of accelerometers to patients receiving hemodialysis could enable widespread assessment of physical resilience in older adults.

Frailty, which can be identified by the presence of at least three of five key indicators as outlined in the Fried frailty index (weight loss, exhaustion, weakness, slowness, and low physical activity level) (6), is another underassessed determinant of patient resiliency. Kutner et al. describe their study of frailty status evolution over a two-year follow-up period of patients ≥ 65 years old receiving hemodialysis in the U.S. A total of 131 participants underwent yearly frailty assessments based on the Fried frailty index, in addition to self-reported indicators of the 4Ms. While most participants had the same frailty status at baseline and at the 24-month follow-up period, 11% of participants had improvement in functionality, whereas 24% had worsening frailty status. Participants who were stable and “robust” were, in general, younger and demonstrated higher 4M-related scores (health status vitality, physical function, and cognitive function).

In a separate manuscript, Thind et al. highlight the importance of frailty in the experience of patients undergoing kidney transplantation, as assessed in the Kidney Transplantation in Older People (KTOP) prospective study of dialysis patients ≥ 60 years old considered for transplantation in the U.K (7). Frailty, as measured by the Edmonton Frail Scale, was assessed in parallel to patient experience and quality of life. Worse frailty scores, particularly related to psychosocial domains, were associated with poor quality of life and patient experience. The authors advocate for assessment of these domains, which can subsequently be targeted in attempts to improve patient experiences and patient-rexperiences.

With these studies and other ongoing research, clinicians increasingly have access to tools to provide age-appropriate healthcare. As more patient-reported data becomes available, how can it be monitored longitudinally? Mobile health (mHealth) platforms, which include any mobile or wireless communication device used to monitor healthcare delivery and patient outcomes, have unique potential in enabling this aspect of patient-centered care. Burrows et al. highlight the ability of mHealth to assist older patients and their care partners in self-care (for instance, setting medication reminders and monitoring symptoms), while allowing providers to remotely access patient data. Such platforms could help increase engagement of patients with kidney disease, which has historically been low (8), in addition to allowing clinicians to serially monitor patient-centered data on what “matters most” to patients during the course of dialysis treatments. mHealth devices have been shown to have high feasibility and acceptability for patients with end-stage kidney disease (9), though additional work is needed to clarify the ideal mHealth structure as defined by relevant stakeholders, and to ensure that mHealth devices can be used by all patients including those with limited literacy.

Overall, these manuscripts contribute valuable insight into methods to deliver person-centered care to older dialysis patients in the Age-Friendly Health System. Clinicians and researchers should continue to work towards identifying tools with high feasibility and acceptability for older patients on hemodialysis, with the ultimate goal of providing 4M-friendly healthcare.

## Author contributions

DP drafted the editorial. All authors contributed to editing and finalization of the manuscript. All authors contributed to the article and approved the submitted version.
